# Effect of temperature on fast transmission of COVID-19 in low per capita GDP Asian countries

**DOI:** 10.1038/s41598-023-48587-3

**Published:** 2023-11-30

**Authors:** Faraz ul Haq, Yasir Abduljaleel, Ijaz Ahmad

**Affiliations:** 1 Centre of Excellence in Water Resources Engineering, University of Engineering and Technology, Lahore, 54890 Pakistan; 2 Department of Civil Engineering, University of Memphis, Memphis, TN 38152 USA; 3 Department of Civil and Environmental Engineering, Washington State University, Richland, WA 99354 USA

**Keywords:** Environmental impact, Environmental sciences

## Abstract

An abrupt outbreak of COVID-19 caused enormous global concerns. Although all countries around the world are severely affected, developing Asian countries faced more difficulties due to their low per capita GDP. The temperature was considered a leading variable in spreading viral diseases, including COVID-19. The present study aimed to assess the relationship between temperature and the spread of COVID-19, with a focus on developing Asian countries. In a few Asian countries, COVID-19 spread rapidly in the summer, while in some countries, there is an increase in winter. A linear correlation was developed between COVID-19 cases/deaths and temperature for the selected countries, which were very weak. A coefficient of determination of 0.334 and 0.365 was observed between cases and average monthly max/min temperatures. A correlation of R^2^ = 0.307 and 0.382 was found between deaths and average max/min monthly temperatures, respectively. There is no scientific reason to assume that COVID-19 is more dominant at low than high temperatures. Therefore, it is believed that the results may be helpful for the health department and decision-makers to understand the fast spread of COVID-19.

## Introduction

Pandemic diseases have proved to be the most distressing in the history of mankind. For instance, the black death pandemic affected one-third of the population of Europe^1–3^, and the Spanish flu casualties more human beings than the entire victims of the first world war^4–7^. Recently, an abrupt epidemic outbreak of COVID-19 disease in 2019^8^ has caused enormous global concerns within the scientific community and healthcare officials^8–10^. COVID-19 disease results from severe acute respiratory syndrome (SARS-CoV) and is usually transmitted through human droplets produced by coughing, sneezing, or talking^11^. More than 77.04 million people have been affected worldwide due to this outbreak (https://covid19.who.int/). These numbers are increasing exponentially daily, with the worst-case scenarios observed in the USA, India, Brazil, the UK, Italy, etc.

The outbreak of COVID-19 led to the disruption of the global economy^12^. Global per capita GDP fell by 4% on average^13^. This pandemic also affected the quality This pandemic also affected the sustainability of life was also impacted by this pandemic^14^. The impact of COVID-19 on GDP was also investigated by^15^ which showed that the average per capita GDP decreases due to the COVID-19 outbreak. Although all countries across the globe are severely affected by the COVID-19 pandemic, developing Asian countries face more difficulties due to their low per capita GDP, fewer healthcare facilities, and large population^16^. The COVID-19 pandemic has slowed down the GDP, along with major economic indicators. In Pakistan, tens of millions of people lost their jobs.

Moreover, this pandemic significantly affects Bangladesh's population, and the 0.89 million population is at job risk. In Afghanistan, the people faced food insincerity, the country is still struggling from economic recession^17^. Nepal's tourist industry was also affected due to this pandemic^18^. India's economy is also affected; its growth rate dropped below 3.1%^19^.

Asian countries, particularly Afghanistan, Yemen, Syria, Nepal, Tajikistan, Kyrgyzstan, Cambodia, Bangladesh, Myanmar, and Pakistan, have very low per capita GDP and are one of the world's poorest regions. About one-third of the world's population lives in this region. Before the outbreak of this pandemic, people in these countries were moderately or severely food insecure and lived below the poverty line^16,20,21^ as most populated cities in the world like Mumbai, Delhi, Karachi, and Dhaka lie in these regions. The rapid increase in COVID-19 cases has affected the economy of these countries badly, and this situation is likely to increase further in the future^22^.

Human beings are already facing global challenges of water scarcity and climate change and are coming under the threat of the COVID-19 pandemic^23,24^. Several regions in Asia are experiencing climatic change, e.g., floods in Bangladesh and India, heavy rainfall events in Nepal, and receding water tables in Pakistan and India^25^. These climatic events have caused an exponential increase in the associated damage. Different studies were conducted to study the impact of meteorological conditions, i.e., relative humidity, sunshine hours, and temperature, on the spread of COVID-19^26–29^. These studies showed that high humidity can increase the spread of COVID-19, while some studies showed that low temperature resulted in the fast transmission of COVID-19. Also, the city's lockdown resulted in improved air quality^30^.

According to most previous studies, climatic conditions, particularly temperature, were considered the leading variable in the spread of viral diseases, including COVID-19 worldwide^31–35^. Different studies have been conducted to assess the impact of temperature on the spread and outbreak of COVID-19^36–39^; however, the relationship between temperature in Asian countries and the COVID-19 pandemic is yet to be understood. Asian countries with low per capita GDP were selected, as these countries were already under stress due to low per capita GDP. There is a need to develop a prediction system for the COVID-19 pandemic^30^. Therefore, the present study aimed to assess the relationship between temperature and the spread of COVID-19, focusing on developing Asian countries. The results of this research may help better understand the linkages between two global crises of climate change and COVID-19, and address the challenges associated with it for sustainable economic development.

## Materials and methods

### Study area

**Figure 1:**
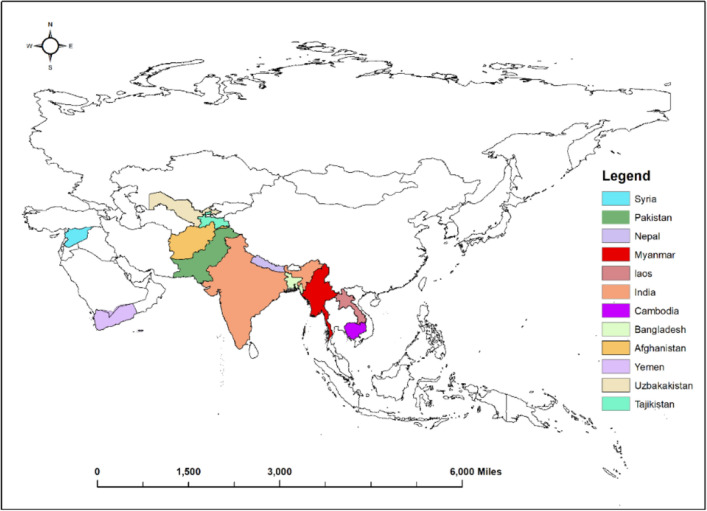
Map of the study area (Software used: ArcGIS Pro).

**Figure 2:**
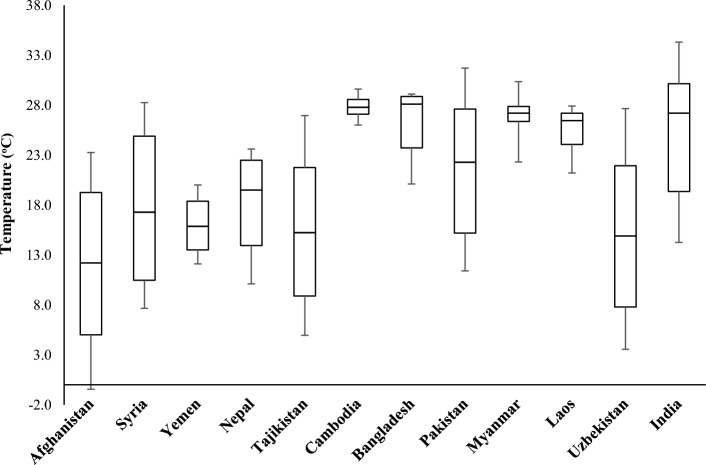
Average annual temperatures of countries.

The study envisaged twelve (12) countries (Fig. 1), including West Asian countries, i.e., Syria and Yemen; South Asian countries, i.e., Afghanistan, Nepal, Bangladesh, Pakistan, and India; Central Asian countries, i.e., Tajikistan, Uzbekistan, and Southeast Asian countries, i.e., Cambodia, Myanmar, and Laos. The variations in the mean annual temperatures of these countries are presented in Fig. 2.

### Data collection

Data of COVID-19 cases was obtained from COVID-19 source data^40^. Average monthly temperature data was obtained from^41^. The countries for the study were selected based on nominal per capita GDP in USD. The GDP of developing Asian countries in the study area is shown in Table 1. These countries were divided into two groups based on low and high temperatures observed during the year. The first group contains six (06) countries with low temperature, i.e., Syria, Yemen, Afghanistan, Nepal, Tajikistan, and Uzbekistan. In contrast, group two contains countries with high temperatures, i.e., Pakistan, India, Bangladesh, Cambodia, Laos, and Myanmar.

**Table 1:** Low GDP nominal per capita Asian countries^42^.

Sr. No	Country	GDP nominal per capita (USD)	Population density (P/km^2^)
1	Laos	2670	32
2	Bangladesh	2195	1265
3	Syria	2032	95
4	India	1876	464
5	Uzbekistan	1831	79
6	Cambodia	1620	95
7	Pakistan	1388	287
8	Myanmar	1244	83
9	Nepal	1116	203
10	Yemen	943	56
11	Tajikistan	877	68
12	Afghanistan	513	60

**Figure 3:**
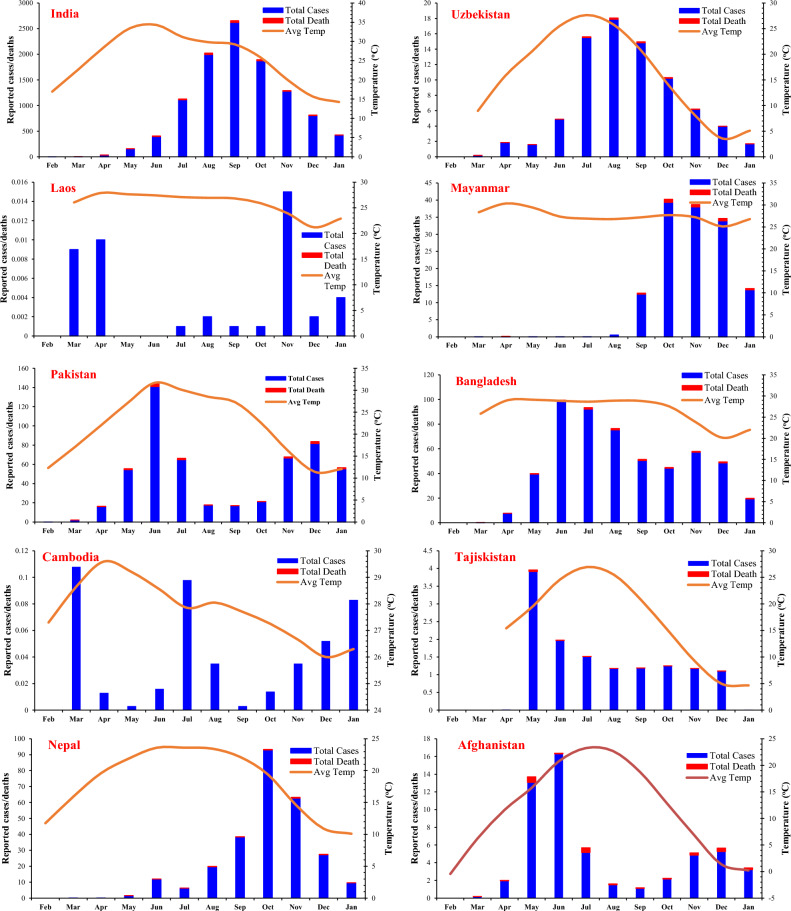

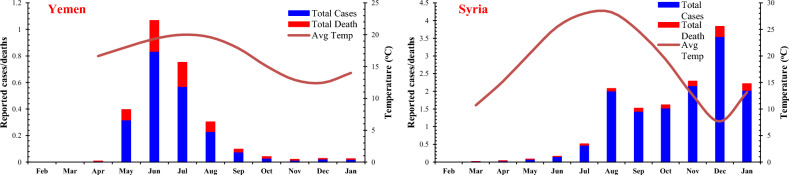
Total reported cases and deaths per month (in Thousands) with averaged monthly temperature.

The graphs of total cases and deaths per month were plotted with an average monthly temperature of COVID-19 reported cases for each low-GDP Asian country, as shown in Fig. 3. Average monthly temperature data was plotted on the secondary axis.

### Statistical analysis

The coefficient of determination (R^2^) was calculated to analyze the association of regional climatological parameters (averaged monthly maximum and minimum temperature) with the total monthly reported COVID-19 cases and total monthly deaths. The coefficient of determination (R^2^) showed vast variations for different data sets. Regression analysis was also applied to evaluate the correlation of reported cases and deaths for the summer and winter seasons. In the summer, months from March to August were considered, while the winter season includes the months from September to February. Correlation between reported cases and deaths was developed for countries where there is a trend of increase in reported cases and deaths during low temperatures, and countries with more reported cases and deaths during high temperatures both for the summer and winter seasons.

## Results and discussion

The data on COVID-19 cases reported in selected Asian countries showed different trends for different regions. Few countries like Syria, Nepal, Myanmar, and Laos showed an increase in COVID-19 cases and deaths during the winter, while in some countries like Yemen, Tajikistan, Bangladesh, Uzbekistan, and India, a trend of increase in reported deaths and cases was observed in the summer. In Afghanistan, Pakistan, and Cambodia, an increase in reported cases and deaths during peak summer (June and July) and winter (November and December) was observed.

The countries found to be more sensitive to the winter season are Syria, Nepal, Myanmar, and Laos. In Syria, there were a maximum of 27.314 cases per million population, with a death rate of 1.943 per million population during the summer (average temperature 28 °C) whereas a significant increase during winter season 123.367 and 202.678 cases per million with 7.37 and 16.799 deaths per million population were observed during November and December with an average temperature of 12.6 °C and 7.65 °C, respectively. In Nepal, cases, and deaths per million population during the winter (14.65 °C) were almost six times higher than the reported cases and deaths during the summer (23.6 °C). Reported cases and deaths in Myanmar during the winter were disastrous. A significant increase in deaths and reported cases was observed during winter. An increasing trend in reported cases in Laos was not devastating. However, an increase in reported cases was observed in the winter during November (21.2 °C).

An increase in reported COVID-19 cases and deaths per million population during the summer season was observed in Yemen, Tajikistan, Bangladesh, Uzbekistan, and India. The reported cases and deaths in Yemen and Tajikistan during May, June, and July showed a significantly increasing trend compared to the reported cases and deaths during winter. In Bangladesh, there was an increase in COVID-19 cases and deaths during June, July, and August (average temperature 28.9 °C), which continued to decline in winter (20.1 °C). In Uzbekistan and India, significant cases and deaths were reported during the peak summer season (29.25 °C), with a decrease in the winter season.

Pakistan, Afghanistan, and Cambodia showed different trends from other countries. There was an increase in COVID-19 cases and deaths during peak summer and peak winter periods. In Afghanistan, the number of cases and deaths rises during May, June, and July, which decreases from August to October. However, an increase in cases and deaths was observed during the peak winter period. The same trend was observed in Pakistan, with a significant increase during the peak summer (June and July) and in the peak winter (November and December). In Cambodia, the highest reported cases were in the peak summer period (27.85 °C) and peak winter periods during November and December.

**Figure 4:**
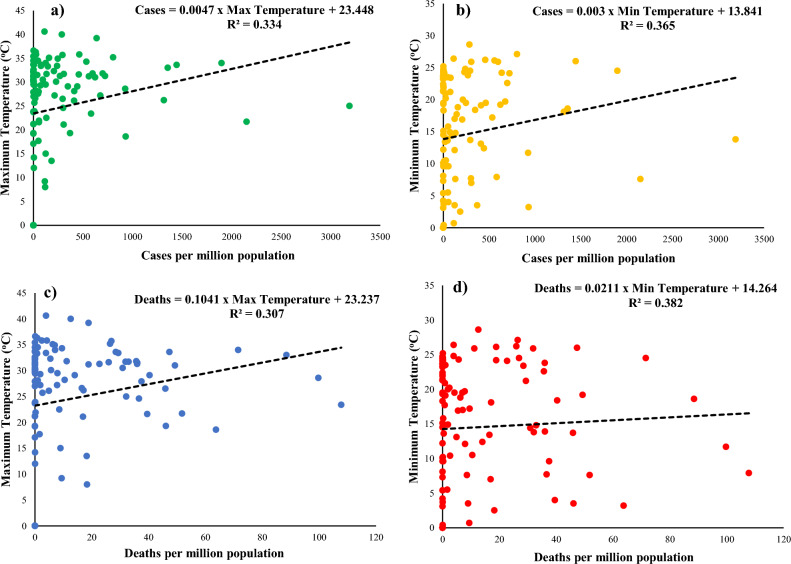
Correlation between (**a**,**b**) max/min temperature and Cases per million population (**c**,**d**) max/min temperature and deaths per million population.

A linear correlation was developed between COVID-19 cases/ deaths and maximum/ minimum temperature for all countries, which were found to be very weak. A correlation of R^2^ = 0.334 and 0.365 between reported cases and mean maximum and mean minimum monthly temperature, respectively. A correlation of R^2^ = 0.307 and 0.382 was found between reported deaths and mean maximum and mean minimum monthly temperatures, respectively, as shown in Fig. 4.

**Figure 5:**
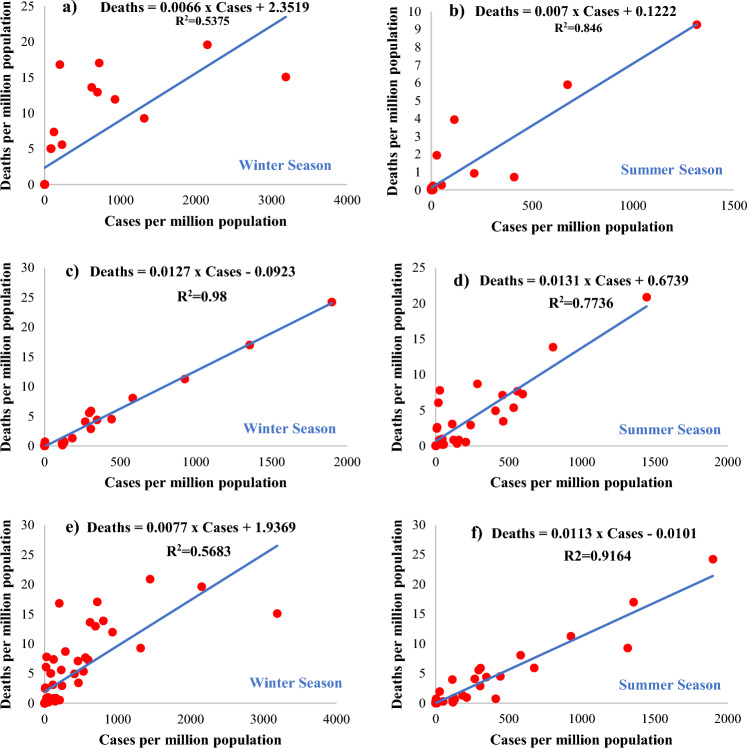
Correlation between deaths vs cases per million population during winter and summer season for (**a**,**b**) Countries showing an increase in COVID-19 during winter season (**c**,**d**) Countries showing an increase in COVID-19 during summer season (**e**,**f**) all countries combined.

Moreover, a linear correlation was developed between reported cases and deaths per million population for the countries, showing an increase in COVID-19 cases during the summer, winter, and peak summer, and winter seasons with the help of the coefficient of determination model. Reported cases in Syria, Nepal, Myanmar, and Laos showed a correlation of 0.5375 and 0.846 with deaths during the winter and summer seasons, respectively. However, in Yemen, Tajikistan, Bangladesh, Uzbekistan, and India, a correlation of 0.98 and 0.7736 was found between cases and deaths during the winter and summer, respectively. The correlation of 0.5683 and 0.9164 was determined during the winter and summer seasons for all the countries combined, as shown in Fig. 5.

The above results showed that the COVID-19 outbreak showed different trends during the summer and winter seasons. Some countries have increased COVID-19 cases during the summer, while others have a rapid outbreak during the winter. The linear correlation between maximum/minimum temperature and cases is 0.334–0.365, which is weak and shows no correlation between temperature and cases. A weak correlation between maximum/minimum temperature and deaths can also be observed. This correlation is not sufficient to prove the association of COVID-19 with temperature.

Population density can be a factor in the spread of COVID-19 in countries like India and Bangladesh. Pakistan and Nepal. Moreover, fewer healthcare facilities and improper lockdowns in developing countries, particularly Afghanistan, Laos, and Myanmar, are significant factors in the COVID-19 outbreak. The increase in cases and deaths was also observed in those months where COVID-19 SOPs were not properly followed.

The above discussion is supported by the fast spread of the novel COVID-19 MERS-CoV in Riyadh during the peak summer season despite high temperatures^43^; however, few studies showed negative relationships between temperature and COVID-19 cases^44^. Jahangiry et al.^45^ also concluded that there is no relationship between COVID-19 cases and the temperature.

## Conclusions

This study investigated the effect of temperature on the spread of COVID-19 in low per capita GDP Asian countries. Few countries exhibited a fast spread of COVID-19 during the summer, and the rest showed an increase during the winter. The results indicated that there is no evidence that the number of people affected with COVID-19 depends on the variations in temperature in different regions. Therefore, no scientific reason was found to assume that COVID-19 is dominant at lower temperatures than the high temperature for these countries. The fast spread of COVID-19 at high and low temperatures may depend on the different variants of the virus, population density, living styles, etc.

This study suggested that proper management, such as social distancing and hand washing, may also slow down the spread of the COVID-19 virus. The results of this study may be helpful for the health department and decision-makers to understand the fast pace of COVID-Declarations.

## Data Availability

The datasets used during the current study are available from the corresponding author upon reasonable request.

## Abbreviations

COVID-19: Coronavirus disease 2019
GDP: Gross domestic product
SARS-CoV: Severe Acute Respiratory Syndrome Coronavirus
USD: United States Dollar
